# The importance of missing data in estimating BMI trajectories

**DOI:** 10.1038/s41598-024-68764-2

**Published:** 2024-07-31

**Authors:** Laura A. Gray

**Affiliations:** 1 Division of Population Health, School of Medicine and Population Health, University of Sheffield, Sheffield, S10 2TN UK; 2 Healthy Lifespan Institution, University of Sheffield, Sheffield, S10 2TN UK

**Keywords:** BMI, Longitudinal analysis, Growth mixture model, Missing data, Diabetes, Obesity

## Abstract

Body Mass Index (BMI) trajectories are important for understanding how BMI develops over time. Missing data is often stated as a limitation in studies that analyse BMI over time and there is limited research exploring how missing data influences BMI trajectories. This study explores the influence missing data has in estimating BMI trajectories and the impact on subsequent analysis. This study uses data from the English Longitudinal Study of Ageing. Distinct BMI trajectories are estimated for adults aged 50 years and over. Next, multiple methods accounting for missing data are implemented and compared. Estimated trajectories are then used to predict the risk of developing type 2 diabetes mellitus (T2DM). Four distinct trajectories are identified using each of the missing data methods: stable overweight, elevated BMI, increasing BMI, and decreasing BMI. However, the likelihoods of individuals following the different trajectories differ between the different methods. The influence of BMI trajectory on T2DM is reduced after accounting for missing data. More work is needed to understand which methods for missing data are most reliable. When estimating BMI trajectories, missing data should be considered. The extent to which accounting for missing data influences cost-effectiveness analyses should be investigated.

## Introduction

Body Mass Index (BMI) trajectories are estimated for several reasons. It is important to measure BMI over time rather than at a single time point when assessing health risks^1–3^. A single measure of BMI hides a patients’ history and could mask the fact that they have had a persistently elevated, increasing or decreasing BMI. BMI trajectories are also frequently used in cost-effectiveness analysis (CEA)^4^. Traditionally, CEA assumes a single mean BMI trajectory, for example, by using random coefficients models^5^, but this can minimise heterogeneity between patients. This is one reason that there is a growing literature on the use of growth mixture models (GMMs), a multinomial modelling approach, to estimate BMI trajectories^2,3,6–10^. This method allows a finite number of distinct trajectories to be determined, which can have varying probabilities for different subpopulations^11^. Recent studies found that there are distinct BMI trajectories that individuals follow as they get older^2,3,6,12^.

The majority of previous studies looking at BMI trajectories using GMMs, fail to account for missing data^2,3,6–8,13^. Previous research has explicitly stated that missing data is a limitation, showing that complete case analysis provided different results, suggesting that missing data analysis warrants more investigation^3^. Complete case analysis assumes that missing data is missing completely at random (MCAR). MCAR assumes that missing data is independent of all variables, both those observed in the data, and those that are unobserved. Some studies have used simple imputation^12^ or multiple imputation to impute BMI values where they are missing^10^, but provided no comparison to determine if imputation made any significant difference to their results. Using multiple imputation assumes that data is missing at random (MAR), that is missing data depends on observed variables but is independent of unobserved data. To the author’s knowledge there is no study that estimates BMI trajectories and accounts for missing data under the assumption of missing not at random (MNAR), that is, the missing data is dependent on unobserved data. It is not possible to formally test whether data is MAR or MNAR. Further research is needed to determine whether different missing data assumptions have a meaningful influence on BMI trajectories.

Missing BMI values in GMMs do not necessarily mean that an individual is discarded from analyses. An individual is included in this analysis if they have at least one non-missing BMI value, unless additional inclusion criteria is imposed by the researcher. However, results may be biased if missing BMI values are MNAR. This assumption could influence both the estimation of the trajectories themselves as well as the probabilities of following each trajectory. Missing data can lead to a loss of power which is irreversible and can cause biased estimates and standard errors. Research is needed into how different ways of accounting for missing data influence BMI trajectories, the probabilities assigned to each trajectory and any subsequent analysis, for example, what effect missing data has when these trajectories are used to predict subsequent health.

BMI trajectory has been shown to have a significant influence on the likelihood of type 2 diabetes mellitus (T2DM)^3,6^; in particular, a consistently elevated BMI has been shown to be a leading cause of T2DM^4^. This highlights the importance of investigating BMI over time rather than at a single point in time. However, when investigating BMI over time, missing data can be a serious limitation, particularly in older adults where data is increasingly missing due to morbidity or mortality.

This study will build on previous research in three ways. First, it will estimate BMI trajectories over a 20 year period, extending the time period that previous studies have used. Next, it will explore a range of missing data methods to account for missing BMI values and discuss their benefits and limitations. Finally, it will explore the extent to which the different methods for analysing missing BMI data influence results when used to predict subsequent risk of T2DM.

## Methods

### Data

The English Longitudinal Study of Ageing (ELSA)^14^ contains data on individuals sampled from the Health Survey for England (HSE) between the years 1998 and 2000 if they were 50 years or over on 1st March 2002. Of 18,813 eligible individuals, 11,415 are included as core members or core partners and included in wave 1 of ELSA. In this study, the HSE baseline data is referred to as wave 0. Subsequent waves are those from ELSA, which follow up these individuals.

As part of the ELSA, nurse visits are carried out during waves 2, 4, 6, 8 and 9 and offered to a subsample of participants. As well as collecting other information, nurses measured participants’ height and weight, allowing their BMI to be calculated (kg/m^2^). Each ELSA follow up is around two years after the previous wave, meaning that there are four years between nurse visits until wave eight when there is a following set of nurse visits two years later in wave 9. The data available on BMI spans the years 1998 to 2019. Self-reported T2DM is available in every wave. In accordance with previous literature^2,3,15^, baseline characteristics include age (years), sex (male/female), smoking status (yes/no) and marital status (married or cohabiting/unmarried or separated). Ethnicity is not included because the ELSA sample are predominantly white (> 97%) and so any influence of ethnicity is unlikely to be identified^3^.

### Statistical analysis

This study uses a two-step process combining a GMM with a discrete-time survival analysis (DTSA) in a structural equation modeling framework. This is done in the same way as a previous study^3^, but extends that study by including additional waves of data and considering different methods to account for missing data. The GMM, DTSA and the Bolck, Croon, and Hagenaars (BCH) method used to link the two are described briefly here. Then, the methods for accounting for missing data are subsequently outlined.

#### Standard growth mixture model (GMM)

A ‘standard’ GMM^16^ is used to estimate distinct BMI trajectories in adults over the age of 50. GMMs can facilitate the identification of differences in longitudinal change in BMI among unobserved groups. This approach uses a multinomial modeling approach to determine whether heterogeneity in the population can be explained by a finite number of distinct subgroups or trajectories, often referred to as latent components.

A dependent variable, $y$ representing BMI for patient $i$ at time $t$ and in component $c$ is defined as follows.

$$y_{itc} = \eta_{0ic} + \eta_{1ic} x_{t} + \eta_{2ic} x_{t}^{2} + \varepsilon_{it}$$.

The random coefficients $\eta_{sic} = \eta_{sc} + \xi_{is}$ for the intercept $\left( {s = 0} \right)$, slope $\left( {s = 1} \right)$ and quadratic $\left( {s = 2} \right)$ terms ($\eta_{0ic}$, $\eta_{1ic}$, $\eta_{2ic}$, respectively) have full covariance matrix. Variances of slope and quadratic terms are set to zero ($\xi_{i1} = 0, \xi_{i2} = 0$). Variance of the intercept term is freely estimated by the model. $x_{t}$ are the time scores, which impose the trend. $\varepsilon_{it}$ is normally distributed with zero mean and variance, $\sigma_{\varepsilon t}^{2}$. Variance of BMI is restricted to be time-invariant.

The intercept is influenced by baseline covariates $z$ (age, sex, smoking status and marital status) so that

$$\eta_{0ic} = \mu_{0c} + \gamma_{0} z_{i} + \zeta_{0i}$$

where $\zeta_{0i}$ is normally distributed with a full covariance matrix.

Probabilities of component memberships are estimated using a multinomial logit model so that

$$\Pr \left( {C_{i} = c{|}z_{i} } \right) = \frac{{e^{{z_{i} \beta_{c} }} }}{{\mathop \sum \nolimits_{k = 1}^{4} e^{{z_{i} \beta_{k} }} }}$$

where $z$ is the same set of baseline covariates that influence the intercept and $\beta$ is a set of estimated coefficients.

#### Bolck, Croon, and Hagenaars (BCH) two-step approach

A BCH approach^17,18^ can be used to assess the relationship between the latent trajectories and observed variables. In this case, it is used to assess the relationship between estimated BMI trajectories and the risk of T2DM. The BCH approach uses a weighted ANOVA to provide weights inversely proportional to the error classification probabilities; observations with less classification error are given more weight in the analysis. These weights are then used in the analysis. This approach does not directly assign individual participants to a specific trajectory and so does not allow the outcomes to directly influence the BMI trajectories, minimising the risk of reverse causality. This approach has been shown to be superior to other approaches that do assign individuals to specific trajectories^19^.

Within the two-step process, step one is the estimation of the BMI trajectories (using GMM) and step two is the estimation of the risks of T2DM using BMI trajectory as a predictor (using DTSA). Previous literature describes the benefits of this approach over alternative approaches^3,19^.

#### Discrete time survival analysis

The hazard function assumes proportional hazards and varies by individual $i$ and component $c$ so that

$$h_{ic} = \frac{1}{{1 + {\text{e}}^{{ - \left( {\beta_{c} + \kappa_{c} v_{i} } \right)}} }} = P(u_{itc} = 1|v_{i} )$$

where $\kappa$ is a set of coefficients and $v_{i}$ is a set of time invariant covariates for individual $i$. $u_{itc}$ is a set of binary variables where, for individual $i$, in component $c$, at time $t$, it is $0$ if no T2DM is present, $1$ if an individual is diagnosed at time $t$ and missing if previously diagnoses.

The time invariant covariates $v_{i}$ include age at baseline and sex. Only these two variables were included in order to give a basic overview of the differences between trajectory groups, whilst also keeping the model parsimonious, allowing the results to be compared to previous research^3^. Note that, because this is a two-step model, the coefficients ${v}_{i}$ in the DTSA (step two) are not directly related to the latent component membership as they are in the GMM (step one).

#### Missing data methods

Although individuals were not removed from the analysis as long as they had at least one valid BMI value, the standard model, described above, assumes that missing data is MAR. The assumption that missing data is MAR is untestable and there is good reason to believe that this assumption may be violated. If BMI measures are MNAR, then the shape of the estimated BMI trajectories may be misleading. This study estimates BMI trajectories using GMMs under different missing data assumptions and compares the trajectories and their influence on T2DM diagnoses.

#### Complete case analysis

First, complete case analysis was performed. This involved removing any individuals which had missing data for BMI at any time point in the analysis. This method has the most restrictive assumptions, assuming that data are MCAR.

#### Multiple imputation

Multiple imputation was used to impute any missing BMI values. This approach assumes that data is missing at random (MAR), that is, the missingness depends on observed variables available in the dataset. BMI at each wave is imputed used available BMI values at other time points, as well as the independent variables using in the model (sex, age, smoking status and marital status) allowing the relationship between these variables to be preserved^20^. The imputed values are random draws from the posterior distributions of the missing values^21^ with 20 imputations. Imputed datasets are analysed using Rubin’s rule^21^.

#### Diggle–Kenward selection model

The Diggle–Kenward selection method^22^ uses repeated measures to predict the probability of missing data at a particular time point. It is a discrete-time survival model for the dropout indicators and is estimated simultaneously alongside the BMI trajectories estimated in the GMM. This approach assumes that data is missing not at random (MNAR) and explicitly models the missingness itself.

Formally, there are $T - 1$ (in this case 5) binary missing data indicators, $d_{1}$,…, $d_{5}$ where $d_{t}$ is $0$ before dropout, $1$ at the first observation after dropout and missing thereafter (*i.e.* all subsequent observations after dropout is first observed.

Dropout due to attrition is influenced by the latent component and by BMI measures from times $t$ and $t - 1$ so that.

$$log\left[ {\frac{{P\left( {d_{it} = 1} \right)|y_{it} ,y_{it - 1} }}{{P\left( {d_{it} = 0} \right)|y_{it} ,y_{it - 1} }}} \right]|_{{C_{i} = c}} = \beta_{0tc} + \beta_{1tc} y_{it} + \beta_{2tc} y_{it - 1}$$.

The logistic regression slopes $\beta$ are allowed to vary by component and by time. Here, BMI is assumed to be MNAR because dropout is related to both past and current BMI. Both ${y}_{it}$ and ${y}_{it-1}$ are observed variables before dropout has occurred and latent variables represented expected BMI after dropout has occurred^23^.

This method for estimating the missingness is estimated jointly with the standard GMM, in effect adjusting the GMM estimates to account for missing data under the assumptions of the Diggle–Kenward method. From here, the BCH approach is used in the same way as for the standard GMM, to incorporate the latent component uncertainty into the DTSA estimation.

#### Roy pattern mixture model

Similar to the Diggle–Kenward model, the Roy pattern mixture model (PMM)^22^ explicitly models missingness and is estimated simultaneously with the GMM. However, this approach assumes that the dropout influences the latent trajectory membership, rather than the latent trajectory influencing the dropout, as the Diggle–Kenward model assumes. Similar to a conventional pattern mixture model, dropout influences the repeated measure (in this case, BMI), but rather than influencing BMI through the random coefficients as a conventional PMM would, the Roy PMM influences them indirectly through component membership.

In this approach, there are $T - 1$ (in this case 5) binary missing data indicators, $d_{1}$, …, $d_{5}$ where $d_{t}$ is 1 for the first observation after dropout and $d_{t}$ is 0 at all other time points.

The probability of component membership depends on baseline covariates, $z_{i}$ and dropout $d_{it}$ at every time point, so that

$$P\left( {C_{i} = c{|}z_{i} ,d_{1i} , \ldots ,d_{Ti} } \right) = \frac{{e^{{z_{i} \beta_{c} + \gamma 0c + \mathop \sum \nolimits_{t = 1}^{T} \gamma_{tk} d_{it} }} }}{{\mathop \sum \nolimits_{k = 1}^{4} e^{{z_{i} \beta_{k} + \gamma 0k + \mathop \sum \nolimits_{t = 1}^{T} \gamma_{tk} d_{it} }} }}$$

replaces the probability equation (Eq. 3) in the standard GMM.

More details on the assumptions made using selection models and pattern mixture models can be found in previous literature along with details on how the missing data is coded for use in a structural equation modelling framework^22,24^.

It is worth noting that additional covariates could be included to predict missingness in the imputation model and in the Diggle–Kenward selection model, but in order not to over complicate the model additional covariates are not included in this example. The different methods of handling missing data, outlined above, are compared to each other, as well as the standard GMM case. The trajectories that are estimated from each analysis are compared, as well as the probabilities of following different trajectories. The differences in predicting the onset of T2DM using BMI trajectory are then investigated.

Data manipulation was performed in Stata (v18)^25^. Data analysis was carried out in Mplus (v8.7)^26^.

## Results

From the 11,415 individuals sampled from the HSE who were considered core members or core partners^27^ at wave 1 of ELSA, 973 individuals were removed because they were younger than 50 years. Of those remaining, 43 had missing values for one or more of the baseline covariates (age, sex, marital status and smoking status) and a further 103 individuals were removed because they had values of BMI considered underweight (BMI < 18.5) in at least one wave, leaving 10,296 individuals. Participants were included in the final sample only if they have at least one valid BMI measure in at least one of the ELSA nurse visits resulting in a further 1077 individuals being removed and a final sample of 9219. Approximately 85% of eligible individuals (those who were interviewed in person) participated in the nurse visits in each wave^14^, although this is not always the same 85% across waves.

Table 1 shows means and standard deviations as well as the number of non-missing observations for each variable within the sample. Mean BMI steadily increased over time and the majority of individuals fell within the range for individuals living with overweight (25 ≤ BMI < 30) at every wave. Around 54% of individuals were female.

**Table 1:** Summary statistics for sample in original data, imputed data and complete case.

	Original data, HSE/ELSA		Imputed data N = 9219	Complete case N = 2002
	Mean (s.d)	#Observed	Mean (s.d)	Mean (s.d)
Age at baseline	61.729 (8.637)	9219	61.729 (8.637)	58.428 (6.391)
	Percentage	#Observed	Percentage	Percentage
Male	45.7%	9219	45.7%	42.9%
Smoker at baseline	17.5%	9219	17.5%	13.5%
Married at baseline	70.2%	9219	70.2%	74.9%
Body mass index (BMI)				
	Mean (s.d)	#Observed	Mean (s.d)	Mean (s.d)
Wave 0	27.688 (4.657)	8558	27.640 (4.700)	27.457 (4.365)
Wave 2	27.895 (4.832)	6537	27.817 (5.013)	27.852 (4.683)
Wave 4	28.204 (5.137)	6717	27.937 (5.251)	28.189 (4.945)
Wave 6	28.217 (5.092)	5588	27.799 (5.323)	28.230 (4.871)
Wave 8	27.918 (5.281)	4676	27.218 (5.536)	27.783 (4.970)
Wave 9	27.587 (5.137)	4045	26.732 (5.479)	27.430 (4.943)
Percentage living with overweight				
	Percentage	#Observed	Percentage	Percentage
Wave 0	45.77%	8558	44.65%	45.90%
Wave 2	43.69%	6537	41.77%	45.70%
Wave 4	43.11%	6717	40.52%	43.71%
Wave 6	43.02%	5588	39.96%	44.31%
Wave 8	40.93%	4676	36.80%	41.91%
Wave 9	41.29%	4045	36.38%	41.81%
Percentage living with obesity				
	Percentage	#Observed	Percentage	Percentage
Wave 0	25.82%	8558	26.04%	23.63%
Wave 2	28.62%	6537	28.92%	27.27%
Wave 4	30.68%	6717	30.74%	30.92%
Wave 6	30.58%	5588	30.42%	30.47%
Wave 8	29.13%	4676	27.91%	27.92%
Wave 9	27.00%	4045	25.08%	26.02%

The complete case analysis resulted in a similar sample to that of the original data. The complete case sample were younger, with fewer males, fewer smokers and more married participants. The mean BMI values were not that different in the complete case analysis compared to the standard GMM, however the standard deviation of BMI values is smaller meaning that participants at both extremes of the distribution may be less likely to remain in the study.

Over 97% of individuals had at least two valid values for BMI during the study period. Table 2 displays the patterns of missing data, which appear for at least 1% of individuals. The largest proportion of individuals (22%) have valid BMI values available for all time points in the models. Interestingly, there is also a large proportion (11%) which have valid BMI values only at baseline and wave 4 (approximately 8 years post-baseline).

**Table 2:** Patterns of missing BMI data.

Percent	Wave 0	Wave 2	Wave 4	Wave 6	Wave 8	Wave 9
Pattern of missing BMI data*						
22%	O	O	O	O	O	O
18%	O	X	O	X	X	X
11%	O	O	X	O	O	O
9%	O	O	O	X	X	X
7%	O	O	O	O	X	X
5%	O	O	X	X	X	X
4%	O	O	O	O	O	X
3%	O	O	X	O	X	X
2%	O	O	X	O	O	X
2%	O	X	O	O	O	O
2%	O	X	X	O	O	O
2%	X	X	O	X	X	X
1%	O	O	O	X	O	O
1%	O	O	X	X	O	O
1%	O	X	O	O	X	X
1%	X	O	O	O	O	O

*All other patterns appear for less than 1% of observations and account for less than 9% of all observations collectively. O—represents observed/non-missing data, X—represents missing data.

### BMI trajectories

The standard GMM model which does not account for missing data gives similar results to a previous study which uses the same methods and data^3^. The present study extends the previous study by including additional waves, namely waves 8 and 9. The trajectories show a similar pattern using the additional waves and the same number of latent trajectories is preferred. Table 3 shows the AIC, BIC and log-likelihoods of standard GMMs estimating BMI trajectories with one to five latent components. The addition of a fifth component made the model struggle to converge; many of the parameters needed to be fixed in order to enable convergence. The additional component also had a very low probability (almost 0%). The BIC and AIC were lowest for the 4 component model. Therefore the 4 component model is chosen to be the preferred model.

**Table 3:** Model fit statistics for the standard GMM of BMI trajectories with 1–5 latent components.

#Components	AIC	BIC	Log-likelihood	Probability of smallest component
1	224,770.487	224,840.464	− 112,376.244	–
2	221,842.941	221,975.118	− 110,904.470	9.5%
3	219,424.115	219,618.493	− 109,687.057	5.8%
4	218,399.149	218,655.729	− 109,166.574	3.7%
5	218,415.149	218,733.930	− 109,166.574	0.0%

**Figure 1:**
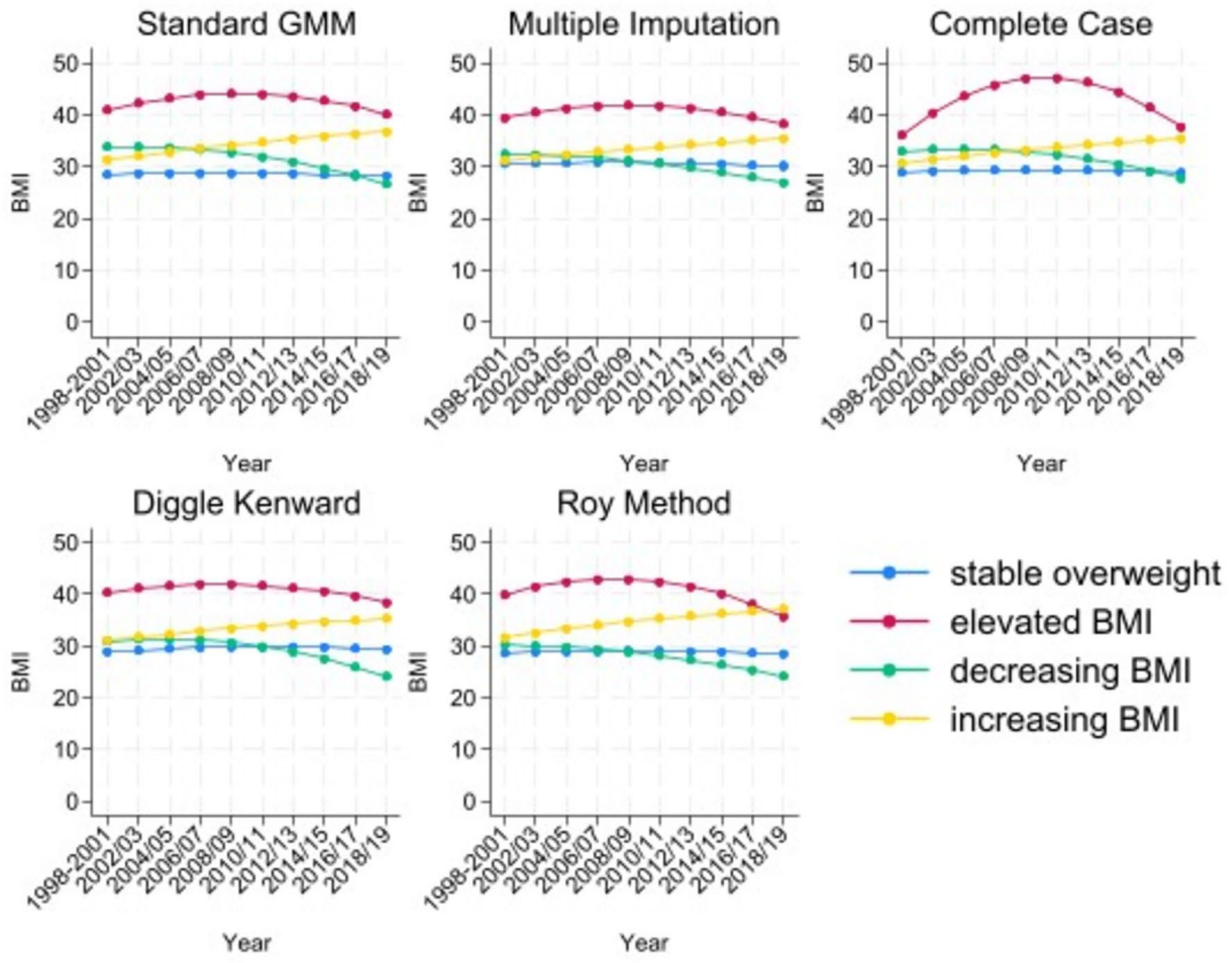
BMI trajectories estimated using standard GMM, multiple imputation, complete case, Diggle–Kenward selection model and Roy pattern mixture model (from top left to bottom right).

Similar comparisons were made across the different methods, with the exception of the complete case analysis where one component in the four component model had a probability of 0.7%. In the multiple imputation model, the 4-component model was selected based on the model fit statistics for each of the 20 imputations. In order to aid comparisons across models, four components are used for each of the different methods and results from this point onwards refer to four component models. Figure 1 shows the estimated trajectories using each of the different methods to account for missing data.

In line with previous literature, the four distinct trajectories are considered to be “stable overweight” (mean BMI consistently around 27); “elevated BMI” (mean BMI consistently high); “increasing BMI” (mean BMI over 30 at baseline and increasing throughout the study period); and “decreasing BMI” (mean BMI around 32 at baseline and reducing to around 25). Within each method, every individual has a positive probability of being in each trajectory.

The multiple imputation and Diggle–Kenward methods produce very similar BMI trajectories; including a flatter elevated BMI trajectory. The multiple imputation, Diggle–Kenward and Roy PMM each have stable overweight, decreasing BMI and increasing BMI trajectories that start at similar baseline BMIs (close to 30). In the complete case analysis, the elevated BMI trajectory is more concave; this is probably due to the small probability of following this trajectory in the complete case analysis. Nevertheless, all methods estimate four trajectories that show the same general pattern.

Table 4 shows the mean probabilities of component membership, for each method, as well as the odds ratios (ORs) for each component by baseline characteristics, again, for each method. ORs are relative to the stable overweight trajectory.

**Table 4:** Probabilities of component membership and odds ratios (ORs) and 95% confidence intervals (CIs) by age, sex, smoking status and marital status.

	Stable overweight (ref)	Elevated BMI OR (95% CI)	Decreasing BMI OR (95% CI)	Increasing BMI OR (95% CI)
Standard GMM	77.7%	3.7%	8.9%	9.7%
Age	1	**0.963** **(0.939, 0.987)**	1.044 (0.997, 1.094)	**0.935** **(0.917, 0.954)**
Male	1	**0.222** **(0.167, 0.297)**	**0.295** **(0.210, 0.415)**	**0.540** **(0.429, 0.681)**
Smoking	1	1.042 (0.778, 1.396)	**1.578** **(1.068, 2.332)**	**2.811** **(2.196, 3.599)**
Married	1	**0.569** **(0.454, 0.714)**	0.873 (0.689, 1.106)	**0.789** **(0.632, 0.986)**
Multiple imputation	57.0%	5.5%	21.0%	16.5%
Age	1	0.991 (0.977, 1.007)	**1.089** **(1.075, 1.103)**	**0.933** **(0.917, 0.949)**
Male	1	**0.295** **(0.217, 0.402)**	**0.720** **(0.622, 0.834)**	0.867 (0.741, 1.015)
Smoking	1	1.305 (0.947, 1.797)	1.110 (0.875, 1.409)	**1.770** **(1.504, 2.083)**
Married	1	**0.731** **(0.555, 0.963)**	1.033 (0.805, 1.326)	0.867 (0.739, 1.017)
Complete case	76.7%	0.7%	9.4%	13.3%
Age	1	0.947 (0.793, 1.131)	**1.099** **(1.064, 1.135)**	**0.946** **(0.918, 0.976)**
Male	1	0.466 (0.076, 2.873)	**0.433** **(0.285, 0.657)**	**0.459** **(0.309, 0.681)**
Smoking	1	1.318 (0.079, 22.047)	**2.829** **(1.651, 4.849)**	**3.638** **(2.122, 6.238)**
Married	1	0.931 (0.045, 19.235)	1.092 (0.699, 1.706)	0.800 (0.538, 1.190)
Diggle–Kenward	39.3%	6.8%	31.2%	22.7%
Age	1	**1.042** **(1.028, 1.057)**	**1.224** **(1.189, 1.261)**	**0.965** **(0.951, 0.980)**
Male	1	**0.269** **(0.217, 0.334)**	1.174 (0.942, 1.464)	**0.818** **(0.703, 0.953)**
Smoking	1	**1.885** **(1.467, 2.422)**	**3.859** **(2.845, 5.235)**	**2.603** **(2.198, 3.083)**
Married	1	**0.536** **(0.442, 0.650)**	0.892 (0.734, 1.084)	0.905 (0.773, 1.059)
Roy model	57.7%	4.6%	31.9%	5.6%
Age	1	1.021 (0.994, 1.049)	**1.130** **(1.101, 1.159)**	**0.960** **(0.941, 0.979)**
Male	1	**0.211** **(0.161, 0.278)**	**0.519** **(0.397, 0.680)**	**0.514** **(0.410, 0.644)**
Smoking	1	1.351 (0.997, 1.830)	**2.559** **(1.875, 3.494)**	**3.127** **(2.409, 4.060)**
Married	1	**0.575** **(0.461, 0.718)**	0.891 (0.695, 1.141)	**0.706** **(0.559, 0.893)**

Significant values are in bold.

The stable overweight trajectory has the highest probability, meaning that individuals are most likely to be on this trajectory. This is the case of each of the methods, but the probabilities do vary substantially across the models. Compared to the standard GMM, all other methods provide a smaller probability of following the stable overweight trajectory, although the complete case method is almost identical (77.8% vs. 76.7%). The multiple imputation method produces a probability of following the stable overweight trajectory of 57.0% compared to 77.7% in the standard GMM. The probabilities for all other trajectories is higher for the multiple imputation method compared to the standard GMM, particularly for the decreasing BMI trajectory, which is 21.0% compared to 8.9% in the standard GMM model. In the complete case analysis, the mean probability of following the stable trajectory is similar to that of the standard GMM, however, the mean probability of following the elevated trajectory is 0.7%, compared to 3.7% in the standard model. In the Diggle–Kenward model, the mean probability of following the stable overweight trajectory is substantially lower than the other models at 39.3%. In the Roy PMM, the mean probability of following the stable overweight trajectory is 57.7%, similar to the multiple imputation model. Both the Diggle–Kenward and the Roy PMM have mean probabilities for the decreasing BMI trajectory that are higher than the other models (31.2% and 31.9%, respective).

In general, the ORs show a similar story across the different methods, and also when comparing to the previous study using data to wave 7^3^. All statistically significant ORs are in the same direction across models, with the exception of the Diggle–Kenward method. Here, increasing age increases the odds of following the elevated trajectory compared to the stable trajectory. A similar result was found using the Roy PMM, but this was statistically insignificant.

Being female, unmarried or a smoker, increases the odds of following the elevated BMI trajectory compared to the stable overweight trajectory. Older age, being female and smoking increases the odds of following the decreasing BMI trajectory. Being younger, female, unmarried or a smoker increases the odds of following the increasing BMI trajectory. Females have a significantly lower probability of following the stable overweight trajectory compared to all other trajectories.

All ORs for the elevated BMI trajectory are statistically insignificant in the complete case analysis where they are statistically significant in other models. The point estimates are generally as expected but they are imprecisely estimated. This is likely to be due to the low probability of following this trajectory when estimated using complete cases.

### Risk of type 2 diabetes mellitus (T2DM)

Table 5 shows the hazard ratios (HRs) for T2DM for each trajectory compared to the stable overweight trajectory, using the BCH weights from the GMMs with different missing data assumptions. Results from the standard GMM are not meaningfully different from those in the previous study, which used data only until wave 7.

**Table 5:** Hazard Ratios (HRs) and 95% confidence intervals (CIs) for diabetes for BMI trajectories compared to stable overweight trajectory, by missing data method.

HR for T2DM* (95% CI)	Stable overweight (reference trajectory)	Elevated BMI HR (95% CI)	Decreasing BMI HR (95% CI)	Increasing BMI HR (95% CI)
Standard GMM	1	**9.091** **(6.536, 12.5)**	**5.263** **(3.300, 8.403)**	**2.03** **(1.898, 3.846**)
Complete case	1	**5.051** **(2.242, 11.364)**	**2.890** **(1.842, 4.525)**	1.116 (0.672, 1.852)
Multiple imputation	1	**5.128** **(3.861, 6.757)**	**2.392** **(1.894, 3.030)**	**1.572** **(1.261, 1.961)**
Diggle–Kenward selection model	1	**5.985** **(3.979, 7.991)**	** < 0.001** **(< 0.001, 0.003)**	2.953 (< 0.001, 13.404)
Roy pattern mixture model	1	**3.908** **(3.002, 4.814)**	**0.401** **(0.285, 0.516)**	**2.021** **(1.647, 2.395)**

*Adjusting for age and sex; T2DM = type 2 diabetes.

Significant values are in bold.

Using the complete case analysis, the HRs for T2DM compared to the stable overweight category are all smaller than they are using the standard GMM analysis but the patterns show the same story. This is also true for the multiple imputation analysis, which provides very similar HRs to that of the complete case analysis.

When using the Diggle–Kenward model or the Roy PMM, the HRs for T2DM for the elevated BMI trajectory compared to the stable overweight trajectory are lower than in the standard GMM, particularly in the Roy PMM. The HR for the increasing BMI trajectory compared to the stable overweight group remains similar in the Roy PMM compared to the standard GMM. It is slightly higher for the Diggle–Kenward model, but this HR is statistically insignificant with very wide confidence intervals.

The HR for the decreasing BMI trajectory compared to stable overweight is substantially lower in both the Diggle–Kenward and Roy PMM. In particular, the Diggle–Kenward model estimates a HR < 0.001 due to estimating a hazard of T2DM of < 0.001 for the decreasing BMI trajectory. This HR is in the opposite direction for the Diggle–Kenward and Roy PMM compared to the other methods, indicating a lower risk for the decreasing BMI trajectory compared to stable overweight, rather than an increased risk based on the other models.

## Discussion

This study estimated five different GMMs, each treating missing data in a different way. The standard GMM ignored any missing data, one used only complete cases, one use multiple imputation to impute missing BMI values and the Diggle–Kenward and Roy PMM explicitly modelled the missingness. The BMI trajectories estimated in these GMMs were then used as predictors in DTSAs to predict HRs for T2DM.

Although the same four trajectories (stable overweight, elevated BMI, decreasing BMI and increasing BMI) were identified using each of the different models, the probabilities of following these trajectories differed substantially between models. This is important because it means that estimated trajectories for an individual could be very different when using the different models.

The complete case GMM has a very similar mean probability for the stable overweight trajectory as the standard GMM, however, the mean probability for the elevated BMI group is much lower in the complete case analysis. This suggests that individuals with missing data had a higher probability of following the elevated BMI trajectory and that when these individuals were removed from the analysis, this trajectory was the most effected.

When BMI values were imputed using multiple imputation, these tended to be BMI values in later waves, more often than earlier waves. Since the mean probability of the stable overweight trajectory is lower and all other trajectories higher in the multiply imputed model than in the standard GMM, this suggests that individuals with more missing values are likely to be those with a higher probability of following the other trajectories, in particular the decreasing BMI trajectory.

Both the Diggle–Kenward Selection model and the Roy PMM directly model the missingness, rather than impute data for missing values. Both provide a smaller probability of following the stable overweight trajectory, compared to the standard GMM, particularly the Diggle–Kenward model. Both estimate mean probabilities for the decreasing BMI trajectory substantially higher than the other models (> 31%). After accounting for dropout, the probability of following a decreasing BMI is substantially increased, again suggesting that individuals with a decreasing BMI may be more likely to dropout.

The multiple imputation model produce HRs for T2DM that are much lower than the standard GMM in all trajectories when comparing to the stable overweight trajectory. This suggests that accounting for missing data reduces the estimated differences in T2DM risk between trajectories and that the standard GMM may be overestimating the hazard in other trajectories or underestimating the hazard in the stable overweight trajectory. Interestingly, the complete case analysis also produces HRs that are much lower than the standard GMM in all trajectories when compared to the stable overweight trajectory.

The Diggle–Kenward and Roy PMM, provide similar results to the complete case and multiple imputation models for the elevated BMI to stable overweight HR. Again, this suggests that the standard GMM is overestimating the HR in this group compared to the stable overweight trajectory. The HR for increasing BMI relative to stable overweight is similar in the Diggle–Kenward, Roy PMM and standard GMM models; this is despite the substantial differences in the mean probabilities of following this trajectory estimated by each of the models. The Diggle–Kenward and Roy PMM, estimate a substantially lower HR than the standard GMM for decreasing BMI verses stable overweight. Moreover, the Diggle–Kenward model estimates a T2DM hazard of almost zero for this trajectory. This could be because the decreasing BMI trajectory has more dropout due to mortality for reasons other than T2DM and if participants that are more likely to follow this trajectory have died then there will be fewer cases of T2DM reported.

The different T2DM HRs found across different missing data assumptions highlights the importance of assessing which assumptions are appropriate in individual contexts. It is not possible at this stage, to choose a preferred missing data method when estimating BMI trajectories, given that different assumptions might be appropriate in different circumstances. The appropriate method of accounting for missing data, or indeed whether it is necessary, will depend on both the data used in estimating the trajectories and the scenario in which the trajectories are used.

The difference in T2DM HRs found in this study also suggests that these assumptions will be important for subsequent CEA that incorporate BMI trajectories in their estimation. Further research is required to determine the extent to which CEAs are influenced by the different missing data assumptions. The results from this study could also apply to estimated trajectories of other health measures; many health trajectories are often included in CEA and missing data is often a problem in their estimation. Results from this study could have substantial implications for a wide range of estimated trajectories used in CEAs.

### Limitations

The main limitation of this study is that it is not known if dropout is due to loss of follow up or death. In a sample of older adults, this is likely to be a greater problem than it would be for the general population. This means that the multiple imputation could be quite misleading, since it will impute BMI values regardless of whether or not a person has died. There is however, no mortality data after wave 6 and so it is impossible to tell whether the participant is missing due to loss of follow up or death. A previous study that investigated the effect of BMI trajectory on mortality using the same data, albeit with fewer waves, found no influence of BMI trajectory on overall mortality^3^. This suggests that the level of mortality does not differ significantly between the distinct trajectories and therefore the influence of any missing data due to mortality is assumed to have minimal effect.

The MNAR models (Diggle–Kenward and Roy PMM) only account for final dropout and not for any intermittent missing data where participants return in a later wave. This could influence the trajectories estimated by these models is the intermittent missing data is not MAR. Future research could investigate this further, potentially by extending the Roy PMM to account for missing data at each time-point.

The ELSA contains information on an overwhelmingly (> 97%) white sample and research using a more diverse sample could shed light on any differences which occur between individuals of different ethnicities.

## Conclusion

Missing data can substantially influence estimations of BMI trajectories. Therefore, when using BMI trajectories to inform subsequent analysis or policymaking, missing data should be considered and accounted for. More research is needed to examine the extent to which accounting for missing data might influence the cost-effectiveness of policies, for example, weight management interventions.

## Data Availability

Data from the English Longitudinal Study of Aging (ELSA) is freely available from the UK data service. More information can be found at NHS Digital. Health Survey for England—Health, social care and lifestyles. 2020 Last edited: 24 August 2020; Available from: https://digital.nhs.uk/data-and-information/areas-of-interest/public-health/health-survey-for-england---health-social-care-and-lifestyles.
